# Nutritional Value of Grain-Based Foods

**DOI:** 10.3390/foods9040504

**Published:** 2020-04-16

**Authors:** Marina Carcea

**Affiliations:** Research Centre for Food and Nutrition, Council for Agricultural Research and Economics (CREA), Via Ardeatina 546, 00178 Rome, Italy; marina.carcea@crea.gov.it; Tel.: +39-06-51494638

**Keywords:** cereals, legumes, pseudocereals, gluten-free grains, macronutrients, micronutrients, bioactives, processing, nutrition

## Abstract

Grains are fundamental in the daily diets of many people worldwide; they are used for the production of popular foods, such as bread, bakery products, breakfast cereals, pasta, couscous, bulgur, and snacks. Botanically, they are the seeds of plants, belonging mainly to the groups of cereals, pseudocereals, and legumes. They contribute macronutrients to the human diet, mainly carbohydrates, but also proteins and lipids, and micronutrients, such as vitamins and minerals. They are also an important source of dietary fibre and bioactives, particularly wholegrains, which are of interest for the manufacturing of high value foods with enhanced health benefits. They can be used for the production of gluten-containing (as well as gluten-free) products. One of the main objectives of the food industry when producing grain-based foods is to manufacture safe, attractive products, with enhanced nutritional value to respond to consumer expectations. The following Special Issue “Nutritional Value of Grain-Based Foods” consists of one review and eight original research papers that contribute to the existing knowledge of important ingredients, such as fat substitutes, and of the technological quality and nutritional role of grains and grain-based foods (gluten-containing and gluten-free), such as bread, muffins, and muesli bars.

Grains are the basis of daily diets for many populations worldwide. They are the seeds of plants, mainly belonging to the botanical groups of cereals, pseudocereals, and legumes.

They contribute macronutrients to the human diet, mainly carbohydrates, but also proteins and lipids, and micronutrients, such as vitamins and minerals. They are also an important source of dietary fibre and bioactives, particularly wholegrains, which are of interest for the production of high value food products with enhanced health benefits [1,2]. Many nutritional guidelines now, in several countries, recommend the inclusion of a greater proportion of wholegrains in the diet for promoting health [3,4,5]. One of wholegrains roles, recently discovered, refers to their prebiotic activity for gut microbiota, which is fundamental for the host’s well-being [6,7]. The content of the aforesaid components varies in grains, depending on genetics and growing conditions, including environment and husbandry.

Humans cannot consume grains in its raw state, so it undergoes a number of processing steps, which might include decortication, dehulling, milling, dough making, extrusion, bread making, couscous making, pasta making, noodle making, bulgur making, etc., up to home cooking [1]. Some grains, thanks to their protein composition, are suitable for the production of gluten-free foods, which are essentially eaten by people suffering from gluten intolerance [8]. Moreover, different kinds of grains can be combined in the same product to take advantage of, in some cases, the complementary composition; thus, producing food with improved nutritional value (see the combination of cereals and legumes that give origin to an excellent aminoacidic composition) [9].

The aim of this special issue was to collect studies on the latest developments in grain science, with regards, in particular, to the improvement of the nutritional value of the raw material due to breeding and/or growing conditions, and the role of processing in keeping or enhancing grains’ nutritional potential for the development of healthy, attractive, and improved products (traditional or new) for human consumption.

The contribution of nine papers in this Special Issue, by 12 research groups, from institutions located in six countries, covers a number of topics connected to the nutritional value of grain-based foods, a very important area in food science. Baked food products, bread and muffins in particular, are the object of research in five papers, whereas gluten-free grains/products are covered by two papers; muesli bars and durum wheat grains are also covered by two articles.

Fat provides important sensory properties, such as colour, taste, texture, and odour to baked food products, which often contain high amounts of fat. There is growing demand by consumers for healthier products with reduced fat content, and manufacturers worldwide have started exploring substitution of fats with so-called fat replacers, which range from complex carbohydrates, gums and gels, whole food matrices, and combinations, thereof. The review by Kathryn Colla, Andrew Costanzo, and Shirani Gamlath summarizes the literature on the effect of fat replacers on the quality of baked food products [10]. The ideal fat replacers for different types of low-fat baked products were a combination of polydextrose and guar gum in biscuits at 70% fat replacement, oleogels in cake at 100% fat replacement, and inulin in crackers at 75% fat replacement. The use of oatrim (100% fat replacement), bean puree (75% fat replacement), or green pea puree (75% fat replacement) in biscuits were equally successful.

Excess sodium intake in the diet is associated with high blood pressure and risk of cardiovascular diseases. Bread has been identified as a major contributor to salt intake in the Italian diet; therefore, the research article by Marina Carcea, Valentina Narducci, Valeria Turfani, and Altero Aguzzi presents a survey of sodium chloride (common salt) content in Italian artisanal and industrial bread, to establish a baseline for salt reduction initiatives [11]. Most of the bread consumed in Italy comes from artisanal bakeries; thus, 135 samples of artisanal bread were sampled in 56 locations from Northern to Southern Italy, together with 19 samples of industrial bread representative of the entire Italian production. Salt content between 0.7% and 2.3% g/100 g (as it is basis) was found, with a mean value of 1.5%, Standard Deviation (SD) 0.3. However, the majority of samples (58%) had a content below 1.5%, with 12% having very low salt content (between 0.5 and 1.0%), whereas the remaining 42% had a salt content higher than the mean value, with a very high salt content (>2.0%) recorded for 3% of samples. With regards to industrial bread, an average content of 1.6% was found, SD 0.3. In this group, most of the samples (56%) had a very high content between 2.0 and 2.5%, whereas 5% only had a content between 1.1 and 1.5%.

Bread is also a very versatile product, which, by adequately changing ingredients, can be tailored to cater for the specific needs of some sectors of the population (e.g., the ageing). The research article by Marina Carcea, Valeria Turfani, Valentina Narducci, Alessandra Durazzo, Alberto Finamore, Marianna Roselli, and Rita Rami explores the effects of functional wheat–lentil bread on the immune functions of aged mice [12]. Legumes are considered excellent ingredients to complement cereal composition, so a functional bread, tailored for the needs of the ageing population, was baked by substituting 24% of wheat flour with red lentil flour, and compared with wheat bread. Its nutritional profile was assessed by analysing proteins, amino acids, lipids, soluble and insoluble dietary fibre, resistant starch, total polyphenols, lignans, and antioxidant capacity (Ferric Reducing Antioxidant Power assay). The wheat–lentil bread had 30% more proteins than wheat bread, a more balanced amino acids composition, almost double the minerals as well as total dietary fibre content, double the amount of polyphenols, higher amounts and varieties of lignans, and more than double the antioxidant capacity. The in vivo effect of 60-day bread consumption on the immune response was studied by means of a murine model of elderly mice. Serum cytokines and intraepithelial lymphocyte immunophenotype from the mouse intestines were analysed as markers of systemic and intestinal inflammatory status, respectively. Analysis of immune parameters in intraepithelial lymphocytes showed significant differences between the two types of bread, indicating a positive effect of the wheat–lentil bread on the intestinal immune system, whereas both breads induced a reduction in serum Interleukin-10.

Bread can also be prepared with gluten-free ingredients, such as corn starch and potato starch. The research group by Przemysław Łukasz Kowalczewski, Katarzyna Walkowiak, Łukasz Masewicz, Olga Bartczak, Jacek Lewandowicz, Piotr Kubiak, and Hanna Maria Baranowska experimented on the substitution of starch with cricket powder as a good source of protein, fat, fibre, and minerals in gluten-free bread [13]. Levels of starch substitutions were 2%, 6%, and 10%; changes caused in the dough rheology and bread texture were studied. While the introduction of cricket powder did not greatly affect dough, the bread was instead characterised by significantly increased hardness and improved consistency. Analyses of water behaviour at the molecular level indicated that cricket powder altered both the bound and bulk water fractions. Moreover, examination of water activity revealed a decreased rate of water transport in samples of bread that contained the cricket powder.

Muffins are also popular bakery products. Generally, they contain high amounts of sugar, and their over-consumption could lead to increased health risks. For this reason, the research group of Jingrong Gao, Xinbo Guo, Margaret A. Brennan, Susan L. Mason, Xin-An Zeng, and Charles S. Brennan studied the potential of modulating reduced sugar (and the potential glycaemic response) in muffins using a combination of Stevia sweetener and cocoa powder [14]. Results illustrate that muffins with 50% replacement of sucrose were similar to the control samples in terms of volume, density, and texture. However, replacement of sugar with 100% Stevia sweetener resulted in reductions in the muffin’s height, volume, and increased firmness (by four-fold) compared to the control sample. Sugar replacement significantly reduced the in vitro predictive glycaemic response of muffins (by up to 55% of the control sample).

Grains, together with a variety of other ingredients, such as fruits, nuts, seeds, and chocolate, are also used for the production of so-called muesli bars, generally consumed as snacks. In dietary guidelines across the world, they are often classified as discretionary food due to their (often) high content of fat and added sugars. A comprehensive nutrition review of grain-based muesli bars in Australia, by means of an audit of supermarket products, is provided by the research article by Felicity Curtain and Sara Grafenauer [15]. Their study aimed to provide a nutritional overview of grain-based muesli bars, comparing data from 2019 with those from 2015. Audits of grain-based Muesli bars were conducted in four major supermarkets in metropolitan Sydney, making up more than 80% of total Australian market share. Mean and standard deviation was calculated for all nutrients on-pack, including whole grain per serve and per 100 g. Compared to 2015, mean sugars declined and 31% more bars were wholegrain. Although categorized as discretionary, there were significant nutrient differences across grain-based muesli bars.

Varieties of gluten-free grains are attracting attention as raw materials to improve the nutritional quality of gluten-free foods and to relieve the monotony of a gluten-free diet. In this regard, the research group of Serena Niro, Annacristina D’Agostino, Alessandra Fratianni, Luciano Cinquanta, and Gianfranco Panfili contributed a research article on gluten-free alternative grains: nutritional evaluation and bioactive compounds [16]. The content of thiamine and riboflavin (water- soluble vitamins) as well as that of carotenoids and tocols (liposoluble vitamins) was determined on nine species of cereals and pseudocereals. The analysed samples showed a high content of bioactive compounds: in particular, amaranth, canihua, and quinoa are good sources of vitamin E, while millet, sorghum, and teff are good sources of thiamine. Moreover, millet provides a fair amount of carotenoids, in particular of lutein.

Data about the nutritional composition of gluten-free products are still limited. For this reason, Idoia Larretxi, Itziar Txurruka, Virginia Navarro, Arrate Lasa, María Ángeles Bustamante, María del Pilar Fernández- Gil, Edurne Simón, and Jonatan Miranda determined the composition of gluten-free breakfast cereals, breads, and pasta. They compared the data with equivalent gluten-containing products and were able to produce a research article on micronutrient analysis of gluten-free products. Their low content was not involved in gluten-free diet imbalance in a cohort of celiac children and adolescents [17]. Micronutrient analytical content differences (minerals and vitamins) were observed in gluten-free products when compared with their gluten-containing counterparts. In order to clarify the potential contribution of the gluten-free products to the gluten-free diet’s micronutrient shortages, analytical data were used to evaluate gluten-free diets in a cohort of celiac children and adolescents. It does not seem that the lower micronutrient content of the analysed gluten-free products contributed to the micronutrient deficits detected in the gluten-free diets in this cohort (whose diets were not balanced). Nevertheless, gluten-free products (fortified for folate and biotin) are proposed to prevent the observed deficiencies.

Durum wheat is the raw material of choice for the production of popular foods worldwide, such as pasta, bread, couscous, and bulgur. With the idea of helping officials set proper quality standards for wholegrain durum wheat flours and products where the germ should be preserved, Valentina Narducci, Enrico Finotti, Vincenzo Galli, and Marina Carcea performed analyses and reported in a research article on lipids and fatty acids in Italian durum wheat (*Triticum durum* Desf.) cultivars [18]. The lipids in the durum wheat grain are, in fact, mainly present in the germ. Samples belonging to 10 popular durum wheat cultivars commercially grown in Italy were harvested and analysed for two consecutive years to account for differences due to changes in climatic conditions. Total lipid content ranged from 2.97% to 3.54% dry basis (d.b.) in the year 2010 and from 3.10% to 3.50% d.b. in the year 2011; the average value was 3.22% d.b., considering both years together. Six main fatty acids were detected in all samples in order of decreasing amounts: linoleic (C18:2) > palmitic (C16:0) ≈ oleic (C18:1) > linolenic (C18:3) > stearic (C18:0) > palmitoleic (C16:1). Significant variations in the levels of single acids between two years were observed for three samples.

The above-mentioned nine papers are the result of a variety of original researches performed worldwide on the general topic of grain science; they provide a valuable overview of current issues, which have attracted attention by the scientific community. They represent state-of-the-art research, provide us with updated knowledge, and give us useful indications on the direction of future research on grain science and technology. For these reasons, this special issue “Nutritional Value of Grain-Based Foods” is worth reading, with much attention, by experts in the field, but also by those who just want to know more about this topic.
